# Mechanistic insight into ligand binding to G-quadruplex DNA

**DOI:** 10.1093/nar/gku247

**Published:** 2014-04-21

**Authors:** Francesco Saverio Di Leva, Ettore Novellino, Andrea Cavalli, Michele Parrinello, Vittorio Limongelli

**Affiliations:** 1 Department of Drug Discovery and Development, Istituto Italiano di Tecnologia, via Morego, 30, I-16163 Genoa, Italy; 2 Department of Pharmacy, University of Naples “Federico II”, via D. Montesano, 49, I-80131 Naples, Italy; 3 Department of Pharmacy and Biotechnology, Alma Mater Studiorum, University of Bologna, via Belmeloro, 6, I-40126 Bologna, Italy; 4 Department of Chemistry and Applied Biosciences, ETH Zurich, and Facoltà di Informatica, Istituto di Scienze Computazionali, Università della Svizzera Italiana, via G. Buffi, 13, CH-6900 Lugano, Switzerland

## Abstract

Specific guanine-rich regions in human genome can form higher-order DNA structures called G-quadruplexes, which regulate many relevant biological processes. For instance, the formation of G-quadruplex at telomeres can alter cellular functions, inducing apoptosis. Thus, developing small molecules that are able to bind and stabilize the telomeric G-quadruplexes represents an attractive strategy for antitumor therapy. An example is 3-(benzo[d]thiazol-2-yl)-7-hydroxy-8-((4-(2-hydroxyethyl)piperazin-1-yl)methyl)-2H-chromen-2-one (compound **1**), recently identified as potent ligand of the G-quadruplex [d(TGGGGT)]_4_ with promising *in vitro* antitumor activity. The experimental observations are suggestive of a complex binding mechanism that, despite efforts, has defied full characterization. Here, we provide through metadynamics simulations a comprehensive understanding of the binding mechanism of **1** to the G-quadruplex [d(TGGGGT)]_4_. In our calculations, the ligand explores all the available binding sites on the DNA structure and the free-energy landscape of the whole binding process is computed. We have thus disclosed a peculiar hopping binding mechanism whereas **1** is able to bind both to the groove and to the 3’ end of the G-quadruplex. Our results fully explain the available experimental data, rendering our approach of great value for further ligand/DNA studies.

## INTRODUCTION

**Figure 1.**
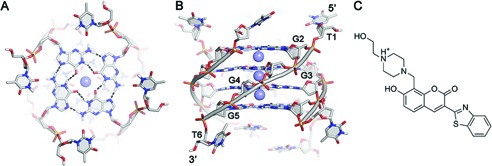
(**A**) Top and (**B**) side view of the crystal structure of the tetrameric G-quadruplex [d(TGGGGT)]_4_ (PDB code 1s45). Nucleotides are shown as sticks colored by atom type and backbone as gray cartoon. K^+^ ions are depicted as purple spheres. Hydrogen bonds are represented as dashed black lines. (**C**) Chemical structure of compound **1** at physiological pH 7.4 (see ‘Materials and Methods’).

Nucleic acids contain the genetic information essential for life. In particular, their sequence encodes vital instructions for the cell, such as replication and transcription. The deoxyribonucleic acid (DNA) usually assumes a standard double helix conformation (1). However, in the last decades, DNA has been found to adopt several alternative conformations having specific roles during cell life. Among these are G-quadruplexes (2), that are stacks of G-quartets, also known as G-tetrads, formed by four guanines hydrogen bonded to each other (Figure 1A) (3). These structures are found in important guanine-rich regions of the human genome, such as gene promoters and telomeres (4–9). In particular, telomeres are ensembles of proteins and noncoding DNA that protect chromosomes termini from unwanted events, such as recombination, degradation, and end-to-end fusion. Furthermore, they prevent chromosomes termini to be recognized as DNA double strand breaks (10,11). Thus, they are crucial for cell lifespan. In normal cells, telomeric DNA shortens at each cell cycle and this eventually leads to senescence or apoptotic cell death (12). In tumor cells instead, telomere length is maintained thanks either to a finely tuned mechanism involving the telomerase enzyme (13) or through recombination events between telomeres, a phenomenon known as the alternative lengthening of telomeres (ALT) mechanism (14).

In 1991, Zahler *et al.* found that folding of telomeric DNA into a G-quadruplex structure inhibits *in vitro* telomerase activity (15), and more recently also the ALT cells have been found to be particularly sensitive to the presence of G-quadruplexes (14). Furthermore, in the last few years, the formation of G-quadruplex structures at telomeres has been found to activate the fast DNA-damage response pathway, inducing rapid apoptosis (16,17). Consequently, molecules able to bind and stabilize G-quadruplex structures can selectively alter telomeric functions in cancer cells, thus leading to a marked inhibition of tumor growth (18,19).

To date, many G-quadruplex selective ligands with potential antitumor activity have been developed (20–27). Most of them, called end-stackers, are usually polyaromatic molecules that bind to the ends of the G-tetrads, such as BRACO-19 (28), RHPS4 (29,30), and telomestatin (31,32). Unfortunately, none of these ligands passed to clinical trials because of their poor drug-like properties and selectivity. At variance with end-stackers, another class of ligands can bind to the grooves of the G-quadruplexes. These compounds, known as groove binders, can be more selective than end-stackers by recognizing the specific groove conformations of different DNA structures. However, despite efforts in developing potent and selective groove binders, only few examples have been to date reported in literature (33–38).

In this framework, a number of structural and computational studies have been performed targeting the telomeric G-quadruplex [d(TGGGGT)]_4_. Although this structure is not a representative human telomeric G-quadruplex, it is often used as a working model for its structural simplicity. In fact, it has a 4-fold symmetry, with all parallel strands, four identical grooves, and three monovalent cations at the center of the guanines stacks (Figure 1A and B) (39). In particular, [d(TGGGGT)]_4_ has been used to identify ligands able to bind both the groove (33,34,36–38) and the stacking surfaces of G-quadruplex (40). One recent example is a structure-based virtual screening campaign that led to the identification of compound **1** (36), which features a 3-(benzo[d]thiazol-2-yl)-2*H*-chrom-en-2-one aromatic scaffold with the positively charged N-(2-hydroxyethyl)piperazinyl branch (Figure 1C). This ligand has a marked binding affinity toward [d(TGGGGT)]_4_ as revealed by nuclear magnetic resonance (NMR) experiments (36). Furthermore, isothermal titration calorimetry (ITC) experiments showed that **1** binds to [d(TGGGGT)]_4_ more tightly than distamycin A (41), that is the reference compound among the G-quadruplex groove binders. These biophysical experiments have been complemented by *in vitro* assays showing that **1** is able to induce DNA damage and cell-cycle arrest in specific cancer cell lines (42).

Despite these efforts, a comprehensive elucidation of the way this compound interacts with DNA is still lacking. In fact, NMR data showed the involvement in the ligand binding of residues both at the groove and at the end of the DNA structure. These observations are suggestive of a complex binding mechanism that was only partially described by the docking calculations where compound **1** was found to bind at the center of the groove (41). Thus, a deeper insight is necessary where considering the full DNA flexibility and the solvent effect during the exploration of the sites available for the ligand binding. These investigations are essential for an exhaustive elucidation of the ligand binding mechanism and to provide the molecular information useful for drug design studies.

With this in mind, we decided to investigate at atomistic level the binding mechanism of compound **1** to its target DNA [d(TGGGGT)]_4_. Unfortunately, the ligand binding and unbinding processes are typically a long time-scale event, in the order of microseconds to milliseconds, difficult to sample with standard techniques, such as molecular dynamics (MD). Thus, the use of enhanced-sampling methods is recommended. Among the emerging techniques, metadynamics (43) has been successful in describing long time-scale biological events and complex ligand binding mechanisms, allowing also to reconstruct the free-energy surface (FES) of the investigated processes (44–48). In the nucleic acids research field, metadynamics has been successfully used to study the unbinding process of small duplex DNA intercalators (49,50), and more recently that of a drug from a protein–duplex DNA complex (51). However, so far this technique has never been applied to investigate the binding of a ligand to a G-quadruplex DNA.

Here, we present a fully unbiased binding study of compound **1** to the G-quadruplex [d(TGGGGT)]_4_ during which no structural information on ligand binding is used. To this goal, we performed well-tempered metadynamics simulations (52), which enhance the sampling allowing an exhaustive exploration of all the possible ligand binding sites on the DNA structure in an affordable computational time. The whole binding event is thus described taking fully into account the DNA flexibility together with the presence of explicit solvent and ions. At the end of the simulation, we reconstructed the free-energy landscape of the binding event together with an accurate estimate of the absolute ligand/DNA binding free energy. These calculations allowed us identifying the main interaction sites of compound **1** and revealing the lowest energy ligand binding modes. In particular, we show that compound **1** interacts with G-quadruplex through a hopping binding mechanism passing from one DNA site to the other. This process is ruled by specific ligand/DNA interactions, such as hydrophobic and polar contacts between the ligand aromatic scaffold and the nucleobases, and salt–bridge interactions between the ligand charged chain and the phosphate groups of the G-quadruplex. Compound **1** shows two lowest free-energy binding modes, being able at the same time to bind to the groove and to the 3’ end of the G-quadruplex. These findings are of great value for the structural optimization of compound **1** and for *de-novo* rational drug design, since different binding modes to G-quadruplex can be exploited to develop new ligands.

Our study also reveals that DNA flexibility plays an important role during the ligand binding and water molecules contribute to stabilize the most relevant ligand binding poses. The results obtained are in agreement with data coming from NMR experiments and allow us to interpret features of the experiments that were previously unclear. For instance, we show that the T6 residue is involved in the ligand binding to the 3’ end of DNA, thus explaining the experimental observations. These findings render our protocol of great value for further investigations on ligand/DNA interaction that can assist drug discovery strategies to develop potent and selective DNA binders.

## MATERIALS AND METHODS

### Molecular docking

Molecular docking calculations of **1** in the crystal structure of the G-quadruplex [d(TGGGGT)]_4_ (PDB code: 1s45) (39) were carried out using the AutoDock4.2 (AD4) software package (53) as implemented through the graphical user interface AutoDockTools (ADT 1.5.4) (54). The ligand tridimensional structure was generated with the Maestro Build Panel (55), and its protonation state at physiological pH (7.4) was assigned using Epik (56). The target DNA structure was prepared through the Protein Preparation Wizard of the graphical user interface Maestro 9.3 (55). Water molecules were removed, hydrogen atoms were added and the co-crystallized Tl^+^ ions were replaced with the more physiologically relevant K^+^ ions. Ligand and receptor structures were converted to AD4 format files using ADT and the Gesteiger–Marsili partial charges were then assigned to the ligand and nucleic acid atoms. In order to allow the ligand to explore the whole conformational space, the docking area was centered on the Cartesian coordinates of the center of mass of the G-quadruplex structure and defined by a box large enough to include the whole macromolecule. Thus, grid points of 90 × 90 × 90 with a 0.375 Å spacing were calculated around the docking area for all the ligand atom types using AutoGrid4.2. Thus, 100 separate docking calculations were performed. Each docking run consisted of 10 millions energy evaluations using the Lamarckian genetic algorithm local search method. Otherwise default docking parameters were applied. Docking conformations were clustered on the basis of the root mean square deviation (rmsd) between the Cartesian coordinates of the ligand atoms (cutoff = 2.0 Å) and were ranked based on the AutoDock scoring function (53). Finally, the lowest energy binding pose provided by preliminary docking calculations was selected as the starting conformation for the subsequent metadynamics simulation. However, it is important to underline that the ligand starting conformation does not affect the final results of a well-converged metadynamics simulation, thus we can arbitrarily choose whatever pose predicted by the docking program as starting structure.

Additional docking calculations using the DNA conformation of the free-energy minimum **Aa** obtained from metadynamics simulation were performed applying the same protocol.

Molecular docking calculations on duplex DNA were performed using the structure of the B-DNA dodecamer [d(CGCGAATTCGCG)]_2_ (PDB code: 1bna) (57), which has been reported to be recognized by **1** (42).

### Molecular dynamics

All the simulations were carried out using the standard *parm99* Amber force field for nucleic acids modified using the recently developed *parmbsc0* parameters (58–60). The NAMD 2.8 code (61) combined with the external plugin PLUMED 1.2.2 (62) was used to perform the simulations. The **1**/[d(TGGGGT)]_4_ complex was solvated in a 12.0 Å layer cubic water box using the TIP3P water model parameters (63). K^+^ cations were used to neutralize the system, with three of these ions placed at the center of the G-tetrads. During the simulations, distance constraints were applied between these ions and their coordinating guanine oxygens. Further 11 K^+^ and 11 Cl^−^ ions were added to reproduce the NMR experimental conditions of 70 mM KCl. A cutoff of 10 Å was used for short-range interactions. The long-range electrostatic interactions were computed by means of the particle mesh Ewald method using a 1.0 Å grid spacing in periodic boundary conditions. The SHAKE algorithm was applied to constraint bonds involving hydrogen atoms, with a 2 fs integration time step. Amber charges were applied to the DNA and water molecules, whereas ligand charges were computed using the restrained electrostatic potential (RESP) fitting procedure (64). The ESP was first calculated by means of the Gaussian package (65) using a 6–31G* basis set at Hartree–Fock level of theory, and then the RESP charges were obtained by a two-stages fitting procedure using Antechamber (66). The system was thus equilibrated through 10 ns MD in the isothermal–isobaric ensemble (NPT) at 1 atm and 300 K before running metadynamics simulations in the canonical isothermal-isochoric (NVT) ensemble.

Standard MD simulations to verify the stability of the metadynamics poses were performed in the NPT ensemble at 1.0 atm and 300 K.

### Metadynamics

The estimation *F(s,t)* at time *t* of the FES *F(s)* as a function of the collective variable (CV) *s* was determined by metadynamics (43) in its well-tempered variant (52), using the following formula:

\begin{equation*} F(s,t)=-{T+ \Delta T\over \Delta T}V(s,t), \end{equation*}

where *V(s,t)* is the bias potential added to the system and *T* is the temperature of the simulation. Δ*T* is the difference between the temperature of the CV and that of the simulation. The bias potential is made up by the sum of the Gaussians deposited along the trajectory of the CV. The exploration of the CV space can be increased by tuning Δ*T*. A Gaussian deposition rate of 0.5 kcal/mol/ps was initially used and gradually decreased on the basis of the adaptive bias with a Δ*T* of 2,700 K. Two CVs were chosen to describe the different ligand conformations during the metadynamics run: (i) the distance (*d*) between the center of mass of [d(TGGGGT)]_4_ and that of the 3-benzothiazol-2-yl-chrom-en-2-one scaffold of compound **1**; (ii) the dihedral angle (torsion − *φ*) defined by the major inertia axis of the ligand and that of DNA (Supplementary Figure S1A and Supplementary Table S1). Gaussian widths of 0.23 Å and 0.05 rad were used for the *d* and *φ* CVs, respectively. During the metadynamics simulations, an upper limit constraint at 27.0 Å was applied to the *d* CV to reduce the conformational space to explore in the fully unbound state.

### The reweighting procedure

To identify the main ligand binding sites on the G-quadruplex [d(TGGGGT)]_4_ together with the lowest energy ligand binding conformations, we applied a recently developed reweighting procedure (67). This algorithm allows reconstructing the Boltzmann distribution relative to CVs different from those biased in the metadynamics run. In fact, once the metadynamics simulation is converged, using the newly computed probability distribution, the FES can be reconstructed as a function of the newly selected CVs. In the present case, two CVs were chosen to distinguish the main ligand binding sites on [d(TGGGGT)]_4_: (i) the projection of the center of mass of the 3-benzothiazol-2-yl-chrom-en-2-one scaffold of compound **1** on the major inertia axis of the target DNA (projection on axis—POA) and (ii) the distance of the same center of mass from this axis (distance from axis—DFA) (Figure 3A and Supplementary Table S1). Finally, to identify the lowest energy ligand binding conformations, the FES was computed as function of (i) the POA CV described above, and (ii) a torsion CV defined by the dihedral angle (*ψ*) between the major inertia axis of the N-(2-hydroxyethyl)piperazinyl tail of compound **1** and that of the G-quadruplex (Supplementary Figure S1B and Supplementary Table S1).

**Figure 2.**
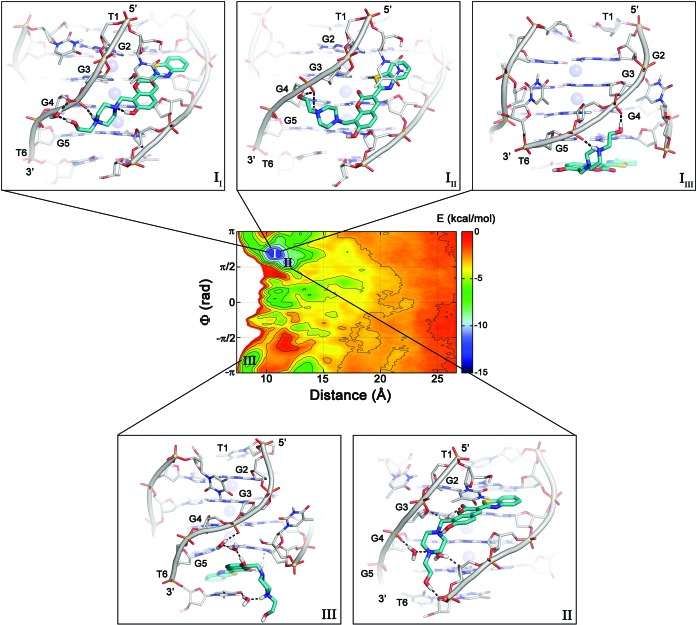
Representation of the binding FES of **1** to [d(TGGGGT)]_4_ as function of the distance (*d*) and dihedral (*φ*) with isosurfaces displayed every 1.5 kcal/mol. The conformations representing the main free-energy minima are shown as insets. One can note that using this FES representation the groove and the 3’-end binding modes are not unequivocally identified by the free-energy minima and the use of more appropriate CVs is necessary (see the main text for details). DNA is displayed as gray sticks and cartoon, while the ligand is shown as cyan sticks. K^+^ ions are depicted as purple spheres. Hydrogen bonds are highlighted as dashed black lines. Nonpolar hydrogens are omitted for clarity.

**Figure 3.**
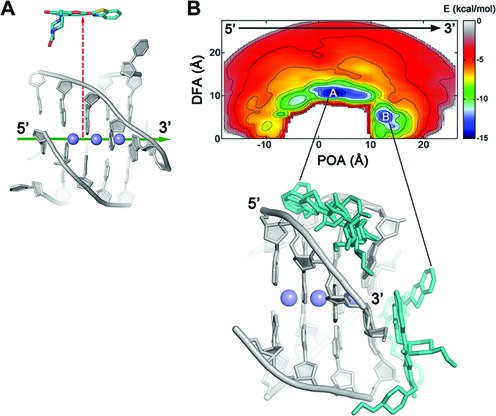
(**A**) Illustration of the “POA” (green arrow) and “DFA” (red dashed arrow) CVs. (**B**) Representation of the binding FES of **1** to [d(TGGGGT)]_4_ as function of the POA and DFA CVs with the most representative ligand binding conformations found at the deepest energy minima **A** and **B**. Isosurfaces of 1.5 kcal/mol are used. DNA is displayed as gray sticks and cartoon, while the ligand is shown as cyan sticks. K^+^ ions are depicted as purple spheres. Thymidines and hydrogens are omitted for clarity.

All figures were rendered with PyMOL (www.pymol.org) and Gnuplot.

## RESULTS

As discussed before, we used well-tempered metadynamics to simulate the binding process of **1** to [d(TGGGGT)]_4_. In any metadynamics simulations, one is required to define a set of properly chosen degrees of freedom, called CVs (52), with whom an adaptive bias is constructed. In such a way, the binding process can be sampled in a relatively short computational time, overcoming large free-energy barriers and reconstructing the FES of the whole binding event (43,52). The choice of the CVs represents a crucial point in this kind of simulations, as they should describe the most relevant slow modes of the investigated process to achieve a reliable estimation of the free energy (68,69).

In the present study, we used a distance (*d*) and a dihedral angle (torsion − *φ*) CV (see ‘Materials and Methods’, Supplementary Figure S1A and Supplementary Table S1). In particular, we purposely chose for *d* the distance between the center of mass of [d(TGGGGT)]_4_ and that of the 3-benzothiazol-2-yl-chrom-en-2-one scaffold of compound **1**. In fact, since [d(TGGGGT)]_4_ presents symmetric binding sites relative to the center of mass of DNA, this choice assigns to these sites the same *d* values, thus considering them equivalent during the simulation. In addition to the distance *d*, the torsion CV describes the different orientations of the ligand relative to DNA during the sampling.

A movie showing the exploration of the DNA binding sites by the ligand during metadynamics simulation can be found as Movie S1.

### The ligand/G-quadruplex binding sites

The whole sampling took ∼1 μs of metadynamics simulations. Looking at the FES computed as function of *d* and *φ* (Figure 2), one can note that while these CVs take into account the 4-fold symmetry of the target and they are able to explore all the binding conformations, they have the disadvantage of not being able to discriminate between the different ligand binding modes (see Figure 2 and Supplementary Discussion for details). Therefore, we decided to reconstruct the free-energy landscape along different CVs using the reweighting algorithm of Bonomi *et al.* (67). This algorithm allows computing the FES as function of CVs different from those biased during the metadynamics simulation. As new CVs, we chose a “POA” and “DFA” CVs, where the axis is the major inertia axis of the G-quadruplex (see ‘Materials and Methods’, Figure 3A and Supplementary Table S1). In such a way, one can discriminate among the different ligand binding sites. The reweighted FES shows that the ligand binds to DNA through a hopping mechanism passing from one binding site to the other. In particular, two main free-energy minima, **A** and **B** were found, with basin **A** approximately 1.5 kcal/mol deeper than **B** (Figure 3B). In **A**, the ligand binds to the groove of the G-quadruplex structure, while in **B** compound **1** stacks at the 3’ region of DNA, thus showing a peculiar dual binding mode. In fact, to the best of our knowledge, no other G-quadruplex ligand has a similar behavior and this information might be of great value for drug design, since one can exploit both binding modes to develop new ligands. From the FES in Figure 3B, one can see that the ligand binds preferably to the 3’ end rather than to 5’. This preference can be explained by the electrostatic properties of [d(TGGGGT)]_4_ and in particular by the electric dipole moment that is oriented toward the 5’ end (see Supplementary Figure S2). The dipole arises from the different topology at the 3’ and 5’ end. In particular, the orientation of the negative phosphate groups, which point toward the 3’ end of the G-quadruplex, favors the binding of **1** to the 3’ terminus.

### The ligand binding modes

**Figure 4.**
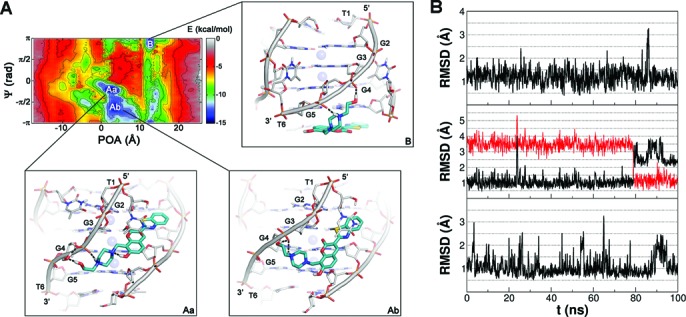
(**A**) Representation of the binding FES of **1** to [d(TGGGGT)]_4_ with the lowest free-energy conformations shown as insets, **Aa**, **Ab**, and **B**. The FES is represented as function of the POA and *ψ* CVs using isosurfaces of 1.5 kcal/mol. DNA is shown as gray sticks and cartoon, while the ligand is shown as cyan sticks. K^+^ ions are depicted as purple spheres. Hydrogen bonds are represented as dashed black lines. Nonpolar hydrogens are omitted for clarity. (**B**) Plots of the average rmsd of the heavy atoms of **1** during over 100 ns of MD simulation with the ligand in the **Aa** (upper), **Ab** (middle), and **B** (lower) binding conformation as starting pose. Upper: the ligand binding mode **Aa** is very stable with a low average rmsd value (1.215 Å) and all the ligand/DNA interactions conserved along the whole simulation, including the water–bridge interaction between the ligand N-(2-hydroxyethyl)piperazinyl branch and the phosphate group of G4. Middle: plot of the ligand rmsd in the binding mode **Ab** relative to its initial pose (black line) and to the **Aa** average conformation (red line). The red plot shows that the **Ab** conformation changes in **Aa** after approximately 80 ns. Lower: the ligand binding mode at pose **B** is very stable with a low average rmsd value (1.076 Å) conserving all the ligand/DNA interactions during the whole simulation.

Although this FES representation is a marked improvement over that shown in Figure 2, it does not fully separates the different ligand binding modes. In fact, a cluster analysis of basin **A** and **B** poses shows a high heterogeneity of ligand conformations, suggesting the necessity of using CVs different from POA and DFA to distinguish the various ligand binding modes at the groove and at the 3’ end. To this end, we recomputed the FES as a function of the POA CV and the torsion CV *ψ*. In particular, the latter was purposely chosen to distinguish the different orientations of the N-(2-hydroxyethyl)piperazinyl tail of the ligand relative to the G-quadruplex axis (see ‘Materials and Methods’, Supplementary Figure S1B and Supplementary Table S1). At variance with the previously computed FES, now the free-energy landscape shows three main energy minima, **Aa**, **Ab**, and **B** (Figure 4A), with the lowest energy minimum **Aa** about 0.9 and 1.4 kcal/mol deeper than **Ab** and **B**, respectively. We stress that to obtain the FESs in Figures 3 and 4A we did not perform a new metadynamics calculation, but simply represented in a different and more physically transparent way the sampling data obtained using the variables *d* and *φ*.

### Groove binding

In basin **Aa**, **1** adopts a groove binding conformation in which it interacts mainly with residues G2, G3, G4, and G5 (Figure 4A) on two neighboring strands. In particular, an H-bond is established between the carbonyl oxygen of the ligand coumarin ring and the amino group of G3, while the N-(2-hydroxyethyl)piperazinyl branch of **1** forms a salt–bridge and a water-mediated H-bond with the phosphate group of G4 on the adjacent strand. The presence of this water-mediated interaction was further validated by 100 ns of unbiased MD simulation (Figure 4B), during which the pose resulted stable. Furthermore, it is interesting to note that in the **Aa** pose the conformation of the N-(2-hydroxyethyl)piperazinyl branch is stabilized by an intramolecular H-bond between the 7-hydroxyl group of the coumarin ring and the proximal nitrogen atom of the piperazinyl ring. Furthermore, residue T1 at the 5’ end points down and stacks on the ligand aromatic scaffold establishing favorable π-stacking interactions. This pose is fully in agreement with the NMR experiments showing a major involvement of residues G2, G3, G4, and G5 in the binding of **1**. In particular, the NMR spectra show a marked variation of the protons chemical shifts of these guanines in the presence of the ligand (36,41). Further evidence on the involvement of these guanines in the binding of **1** comes from additional NMR experiments that show the decrease of the chemical shift variations of all the guanines when **1** is made to interact with mutated G-quadruplexes, namely, [d(TGG^Br^GGT)]_4_ and [d(TGGGG^Br^T)]_4_ (41). In this case, the presence of the bulky bromine atom inside the grooves hampers the binding of **1**. Finally, the “sandwich-like” conformation adopted by T1 in basin **Aa** (Figure 4A) is in line with the involvement of this residue in the ligand binding shown by the NMR studies (36,41). It is worth mentioning that the **Aa** pose is similar to one of the two binding modes proposed in previous docking studies (Supplementary Figure S3) (41). However, few differences between the docking pose and **Aa** can be noticed. In particular, the docking binding mode is slightly shifted toward the 5’ end if compared with **Aa**. In fact, at variance with **Aa**, in the pose predicted by docking the N-(2-hydroxyethyl)piperazinyl group establishes favorable contacts with the phosphate group of G2 instead of that of G4. These differences mainly arise from the fact that the docking algorithm used in this study does not use an explicit solvent model and treats DNA as a rigid body. Thus, water mediated interactions and DNA conformational rearrangement to better accommodate the ligand are not described. As a proof of concept, we redid the docking calculations with the same program using the DNA conformation found in minimum **Aa** (see ‘Materials and Methods’). In this test, the docking program finds the metadynamics pose **Aa** to be one of the most favorable binding modes (Supplementary Figure S4). These outcomes confirm the soundness of the previously reported binding mode of **1** but suggest at the same time to take exhaustively into account the target flexibility and solvent effects when more rigorous calculations on ligand binding are needed.

The second energy basin **Ab** (Figure 4A) corresponds to another groove binding conformation in which the ligand aromatic scaffold is slightly shifted toward the 3’ end. At variance with **Aa**, in this pose, the sulfur atom of the benzothiazole moiety H-bonds with the amino group of G3. On the other hand, the N-(2-hydroxyethyl)piperazinyl establishes two polar contacts with the phosphate group of G4 in a way comparable to **Aa**.

To verify the stability of the binding conformations of basin **Aa** and **Ab**, over 100 ns unbiased MD simulations were performed. Standard MD has been indeed successfully used to describe the interactions engaged by ligands locally at the DNA binding site (70). In our simulations, the results show a different behavior in the two cases. In particular, the binding conformation **Aa** is stable during the whole simulation with low ligand rmsd values (Figure 4B), and conserves all the ligand/DNA interactions. In contrast, **Ab** first maintains its original position in the binding site; then, after approximately 80 ns of simulation, it slightly moves toward the 5’ end transforming in **Aa**. This finding is not totally surprising since, looking at the FES in Figure 4A, the energy barrier that separates **Ab** from **Aa** is rather small, approximately 1.5 kcal/mol, and thus it can be overcome in the standard MD time scale. This motion can be appreciated looking at Figure 4B where the rmsd values of the ligand with respect to its position in the **Aa** conformation are shown.

Furthermore, to investigate the G-quadruplex binding selectivity of **1** toward duplex DNA, we performed docking calculations on a B-DNA structure (see ‘Materials and Methods’). The docking results show that **1** binds to the duplex minor groove similarly to what observed for G-quadruplex (see Supplementary Discussion and Supplementary Figure S5). These results confirm the experimental data showing that **1** is able to bind the duplex DNA, interfering with the binding of the known B-DNA ligand distamycin A (41). The structural information coming from these computations can be exploited to develop more potent and selective G-quadruplex ligands.

### End-stacking

At variance with **Aa** and **Ab** where **1** shows groove binding modes, in basin **B**, compound **1** lies at the 3’ end where it engages favorable π-stacking interactions with G5 and close contacts with two neighboring thymine residues (T6; Figure 4A). However, the N-(2-hydroxyethyl)piperazinyl moiety partially inserts into the groove interacting with the sugar-phosphate backbone of DNA. In particular, the N4 atom of the piperazinyl ring salt bridges with the phosphate group of G5, while the hydroxyethyl group H-bonds with the phosphate of G4. It is worth mentioning that NMR data show a marked chemical shift variation of T6–H6 and T6–methyl protons in ^1^H NMR titrations on the **1**/[d(TGGGGT)]_4_ complex (36,41), suggesting a role for T6 in the ligand binding. However, this residue is not involved in the previously reported ligand binding modes, while these data are now for the first time rationalized by our results.

The stability of this binding conformation was checked running an over 100 ns unbiased MD simulation during which the ligand binding mode was stable conserving all the ligand/DNA interactions (Figure 4B).

### DNA-ligand binding free energy

The absolute ligand binding free energy (Δ*G*_bind_) can be computed from the FES as the free-energy difference of the system with the ligand in the bound and unbound state. However, to obtain an accurate estimate of Δ*G*_bind_, a number of recrossing events between these two states should be observed during the simulation (48). In our simulations, the ligand visits several times both the bound (7.0 ≤ *d* ≤ 14.0 Å) and the unbound states (24 < *d* < 26 Å) as shown in Supplementary Figure S6, leading to a quantitatively well-characterized FES. At the end of the simulation, Δ*G*_bind_ is equal to –9.4 ± 1.4 kcal/mol. Unfortunately, experimental data on the binding free energy of this ligand are not yet available. However, the computed value falls in the range from –9.0 to –11.0 kcal/mol, which are typically the experimental Δ*G*_bind_ values measured for lead compounds (71).

To provide a picture of the convergence of the binding free-energy estimation, the free-energy difference between the bound and unbound state was computed as a function of the simulation time (Supplementary Figure S7). This figure shows that after 870 ns the free energy is converged. In fact, for the rest of the simulation the free-energy difference between bound and unbound states does not considerably change, while the ligand continues visiting both the bound and unbound states.

## DISCUSSION

In the present study, we used metadynamics simulations to study the binding process of **1** to the G-quadruplex [d(TGGGGT)]_4_. From the metadynamics calculation, we reconstructed the free-energy landscape of the ligand/DNA binding process, estimating also the absolute DNA/ligand binding free energy. We have thus identified the preferred ligand binding sites on DNA associated with the lowest energy ligand binding modes. In particular, we have shown that **1** interacts with G-quadruplex through a hopping binding mechanism passing from one DNA site to the other. During this process the ligand assumes two lowest free-energy binding modes, one to the groove and the other to the 3’ end of the G-quadruplex. This is a peculiar binding behavior with respect to other G-quadruplex ligands that stimulates further experimental and theoretical investigations. For instance, in drug design one might exploit both the binding modes, through a lead optimization procedure of compound **1**, to develop more potent and selective compounds. Our study also shows that solvent and target flexibility play an important role during the ligand binding and neglecting such aspects might lead to inaccurate results. The relevance of our results is enhanced by the fact that some of the previously reported NMR data (41) are here for the first time rationalized. For instance, we have shown that the T6 residue is involved in the 3’-end binding mode, thus explaining the experimental observations.

Our work is an example of fully unbiased binding study of a ligand to DNA where no structural information on ligand binding is used. Here, metadynamics simulations are applied for the first time to describe the ligand binding to G-quadruplex. In our study, all the possible binding sites on the DNA structure are visited and energetically evaluated. Our findings confirm and well integrate the previously reported experimental data, highlighting the importance of using atomistic simulations to assist, complement, and rationalize experiments on biologically relevant phenomena. The results, achieved at a relatively low computational cost, render our protocol valuable for further investigations on various forms of ligand/DNA interaction and useful even for more automated drug discovery strategies to develop potent and selective DNA ligands.

## SUPPLEMENTARY DATA

Supplementary Data are available at NAR Online.

SUPPLEMENTARY DATA
