# Supplementary material for: Mechanistic insight into ligand binding to G-quadruplex DNA
Source: Nucleic Acids Res. 2014 Apr 21;42(9):5447–55. doi: 10.1093/nar/gku247 (PMC4027208; doi:10.1093/nar/gku247)
Supplement: SUPPLEMENTARY DATA [file supp_42_9_5447__index.html]

SUPPLEMENTARY DATA 

# Mechanistic insight into ligand binding to G-quadruplex DNA

## SUPPLEMENTARY DATA

**Files in this Data Supplement:**

- SUPPLEMENTARY MOVIE
- SUPPLEMENTARY DATA
