# Supplementary material for: Mechanistic insight into ligand binding to G-quadruplex DNA
Source: Nucleic Acids Res. 2014 Apr 21;42(9):5447–55. doi: 10.1093/nar/gku247 (PMC4027208; doi:10.1093/nar/gku247)
Supplement: SUPPLEMENTARY DATA [file supp_gku247_revised_si.doc]

**Supplementary Data**

**Mechanistic Insight into Ligand Binding to G-quadruplex DNA**

Francesco Saverio Di Leva^1^, Ettore Novellino^2^, Andrea Cavalli^1,3^, Michele Parrinello^4^ and Vittorio Limongelli^2,*^

^1^Department of Drug Discovery and Development, Istituto Italiano di Tecnologia, via Morego, 30, I-16163 Genoa, Italy

^2^Department of Pharmacy, University of Naples “Federico II”, Via D. Montesano, 49, I-80131 Naples, Italy

^3^Department of Pharmacy and Biotechnology, Alma Mater Studiorum, University of Bologna, via Belmeloro 6, 40126 Bologna, Italy

^4^Department of Chemistry and Applied Biosciences, ETH Zurich, and  Facoltà di Informatica, Istituto di Scienze Computazionali, Università della Svizzera Italiana, Via G. Buffi 13, CH-6900 Lugano Switzerland

*To whom correspondence should be addressed. Tel: +39 081 678 641; Fax: +39 081 678 107;

Email: vittoriolimongelli@gmail.com

**SUPPLEMENTARY DISCUSSION**

**Binding modes of compound 1 representing the free-energy minima of the FES shown in Figure 2**

Looking at the FES resulting from the metadynamics simulation using as CVs the distance *d* and dihedral angle, *φ*, (Figure 2), three main energy minima can be detected, **I**, **II** and **III**. After clustering the ligand conformations representing the deepest energy minimum, basin **I**, three different binding modes were found, **I_I_**, **I_II_**, **I_III_** (Figure 2). In fact, using this FES representation the groove and the 3’ end binding modes are not unequivocally identified by the free-energy minima and the use of more appropriate CVs is necessary (see Main Text for details). In particular, **I_I_**, **I_II_**, **I_III_** correspond to the **Aa**, **Ab** and **B** poses, respectively, of the FES reported in Figure 4A in the main text. The second energy minimum, basin **II** in Figure 2, is around 2.5 kcal/mol higher than basin **I**. In this minimum, **1** adopts a groove binding conformation establishing several polar contacts with DNA. Here, water molecules mediate the interactions between the carbonyl oxygen of the ligand chromenone ring and the amino group of G3 and between the protonated nitrogen of the ligand piperazinyl ring and two G4 residues. In addition, a H-bond interaction is established by the hydroxyethyl of **1** with the phosphate groups of T6. The two main basins, **I** and **II**, are separated by a low free-energy barrier (~3 kcal/mol from **I** to **II**). It is reasonable to suggest that pose **II** represents the first binding event of **1** to DNA before reaching its final position in the groove. Alternatively, **II** can be considered the first unbinding event of the ligand from the groove of the G-quadruplex. In the third energy minimum, basin **III**, which is around 5 kcal/mol higher than basin **A**, the ligand assumes a 3’ end-stacking binding conformation (Figure 2). However, this pose is rather different from the 3’ end binding conformation described in the main text, which corresponds to pose **I_III_** in Figure 2 and pose **B** in Figure 4A. In particular, while in pose **I_III_** (**B** in Figure 4A) the ligand tail engages direct H-bonds with DNA, in this pose all the polar interactions are mediated by water molecules, such as the H-bond interaction between the ligand piperazinyl ring and the carbonyl oxygen of T6. Due to the weaker ligand/DNA interactions and the higher free-energy value of this minimum, this pose can be considered as a subsidiary binding mode.

**Binding mode of compound 1 to duplex DNA**

To investigate the G-quadruplex groove binding selectivity of **1** towards duplex DNA, we performed docking calculations on a B-DNA dodecamer (PDB code: 1bna) (57) (see Materials and Methods in the main text). In the most populated docking pose, **1** is found to bind to the duplex minor groove where it engages few polar contacts (see Figure S5). In particular, a H-bond is established between the hydroxyl group on the ligand coumarin ring and the phosphate group of C9, while the N-(2-hydroxyethyl)piperazinyl branch of **1** forms a salt-bridge and a H-bond with the phosphate group of T7 on the opposite strand. This groove binding conformation is similar to that found by the docking program for the **1**/[d(TGGGGT)]_4_ complex. These results are in line with the experimental data showing that **1** is able to bind to the duplex DNA, interfering with the binding of the known B-DNA ligand distamycin A (41). The structural information coming from these computations can be used to develop more potent and selective G-quadruplex ligands.

**SUPPLEMENTARY FIGURES**


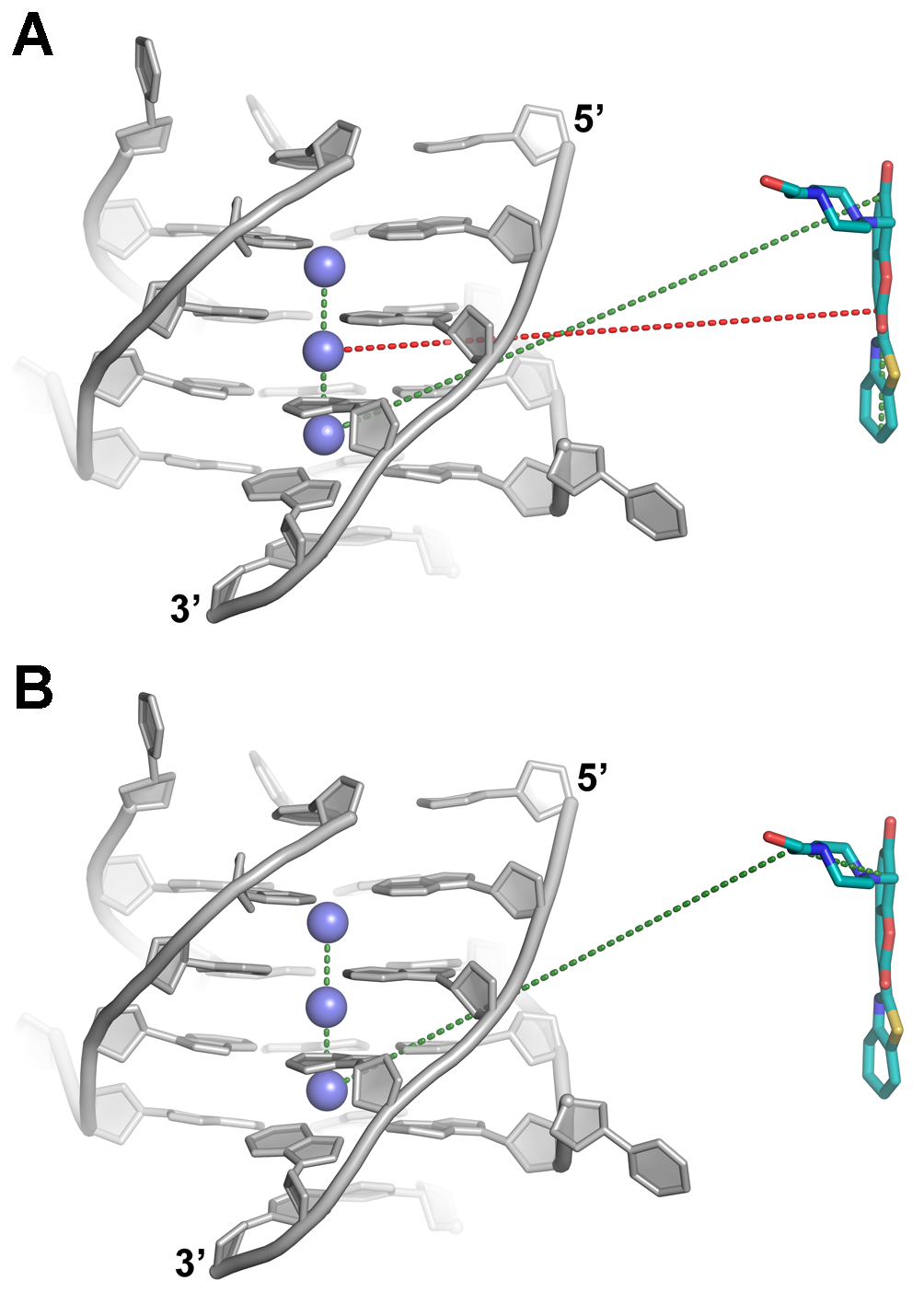


**Supplementary Figure S1.** Representation of some collective variables (CVs) used in the metadynamics study of the **1**/[d(TGGGGT)]_4_ binding process. (**A**) The distance (*d*) between the center of mass of [d(TGGGGT)]_4_ and that of the 3-(benzo[*d*]thiazol-2-yl)-2*H*-chrom-en-2-one scaffold of **1** (red dashes) and the dihedral angle (torsion - *φ*) defined by the major inertia axes of the ligand and that of the target DNA. (**B**) The dihedral angle (torsion - *ψ*) defined by the (N-(2-hydroxyethyl)piperazinyl)methyl tail of the ligand and the G-quadruplex major inertia axis (green dashes), that was used in combination with the POA CV (see Figure 3A) to compute the binding FES shown in Figure 4A.


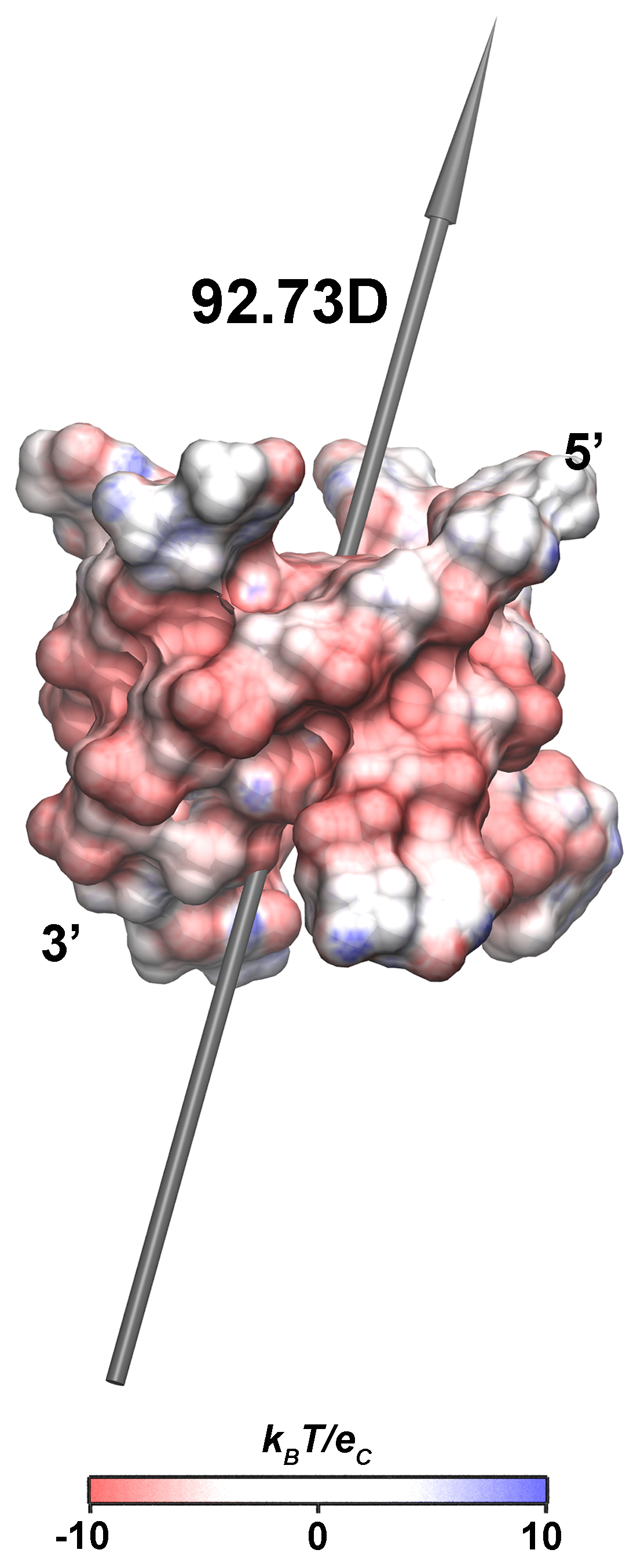


**Supplementary Figure S2.** Electrostatic potential surface and dipole moment (grey arrow) of the G-quadruplex [d(TGGGGT)]_4_. The electrostatic potential was computed using the APBS plugin within VMD 1.9.1. The color scale ranges from -10.00 (red) to 10.00 (blue) *k*_B_T/*e*_c_.

**
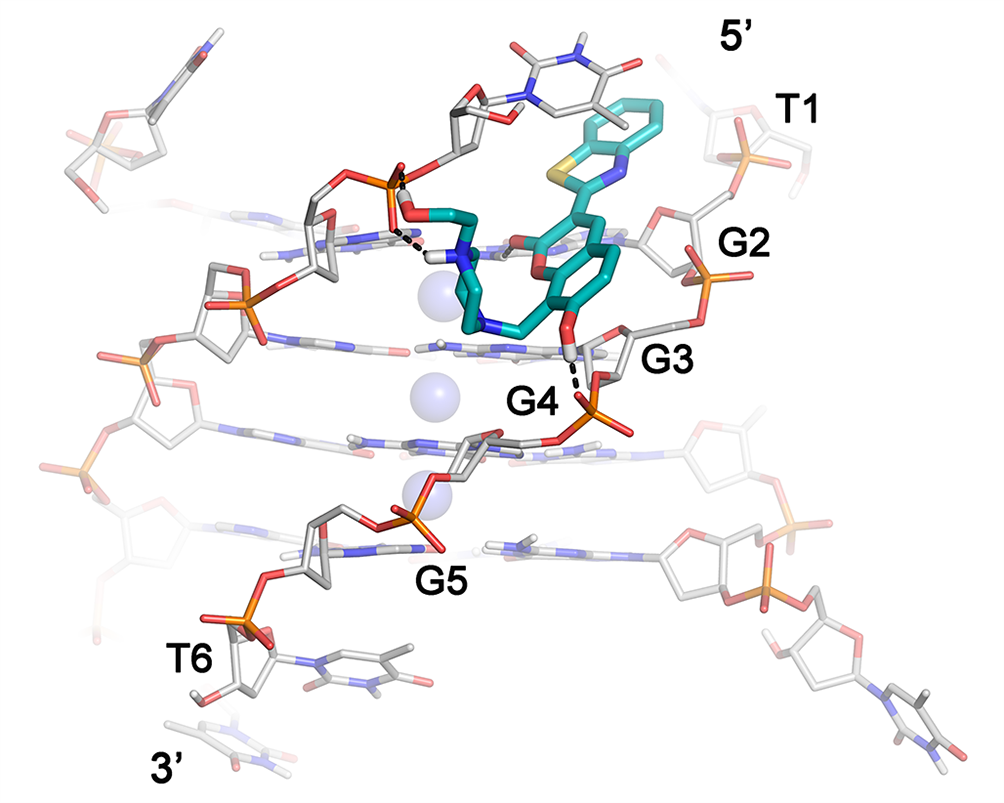
**

**Supplementary Figure S3.** One of the two Autodock docking poses of compound **1** proposed in a previous work (41). DNA is displayed as grey sticks, while the ligand is shown as cyan sticks. K^+^ ions are depicted as purple spheres. Hydrogen bonds are highlighted as dashed black lines. Non-polar hydrogens are omitted for clarity.


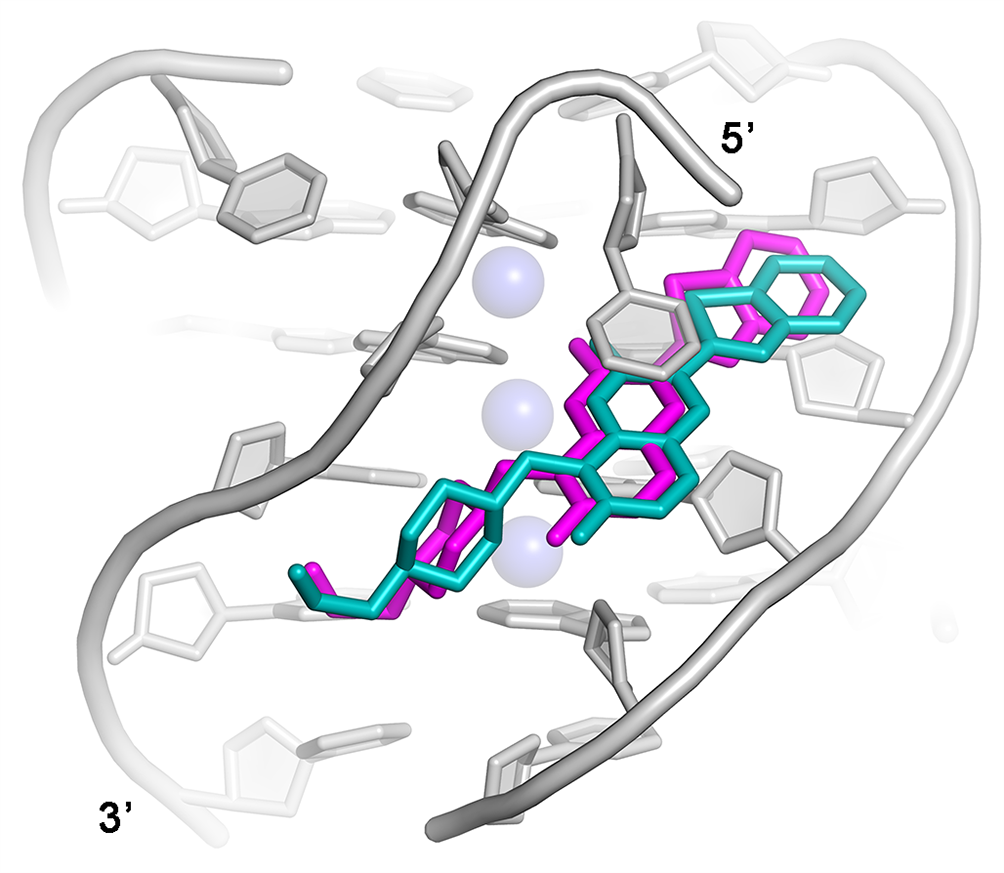


**Supplementary Figure S4.** Overlapping of the **Aa** binding conformation of compound **1** obtained from metadynamics (cyan) and that predicted by AutoDock4.2 (magenta) using the [d(TGGGGT)]_4_ conformation derived from the metadynamics calculation. The DNA is shown as grey sticks and cartoon. Hydrogens are omitted for clarity. A detailed description of the **1/**[d(TGGGGT)]_4_ interactions in the **Aa** conformation is reported in the main text (see the *Groove binding* paragraph in the *Results* section).


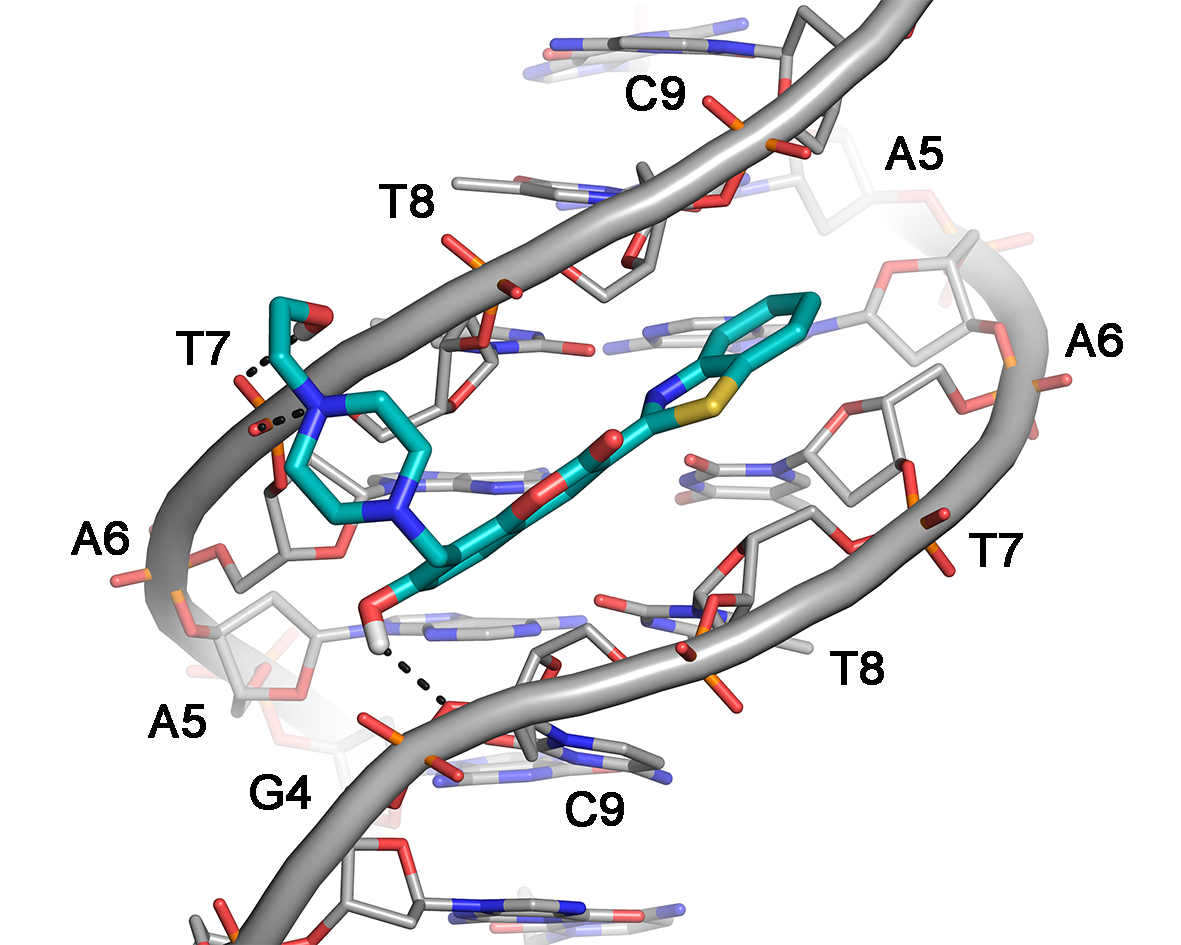


**Supplementary Figure S5.** Docking pose of **1** (cyan sticks) to the B-DNA dodecamer [d(CGCGAATTCGCG)]_2_ (PDB code: 1bna) (57) as predicted by Autodock4.2 (53). The DNA is shown as grey sticks and cartoon. Nonpolar hydrogens are omitted for clarity.

**
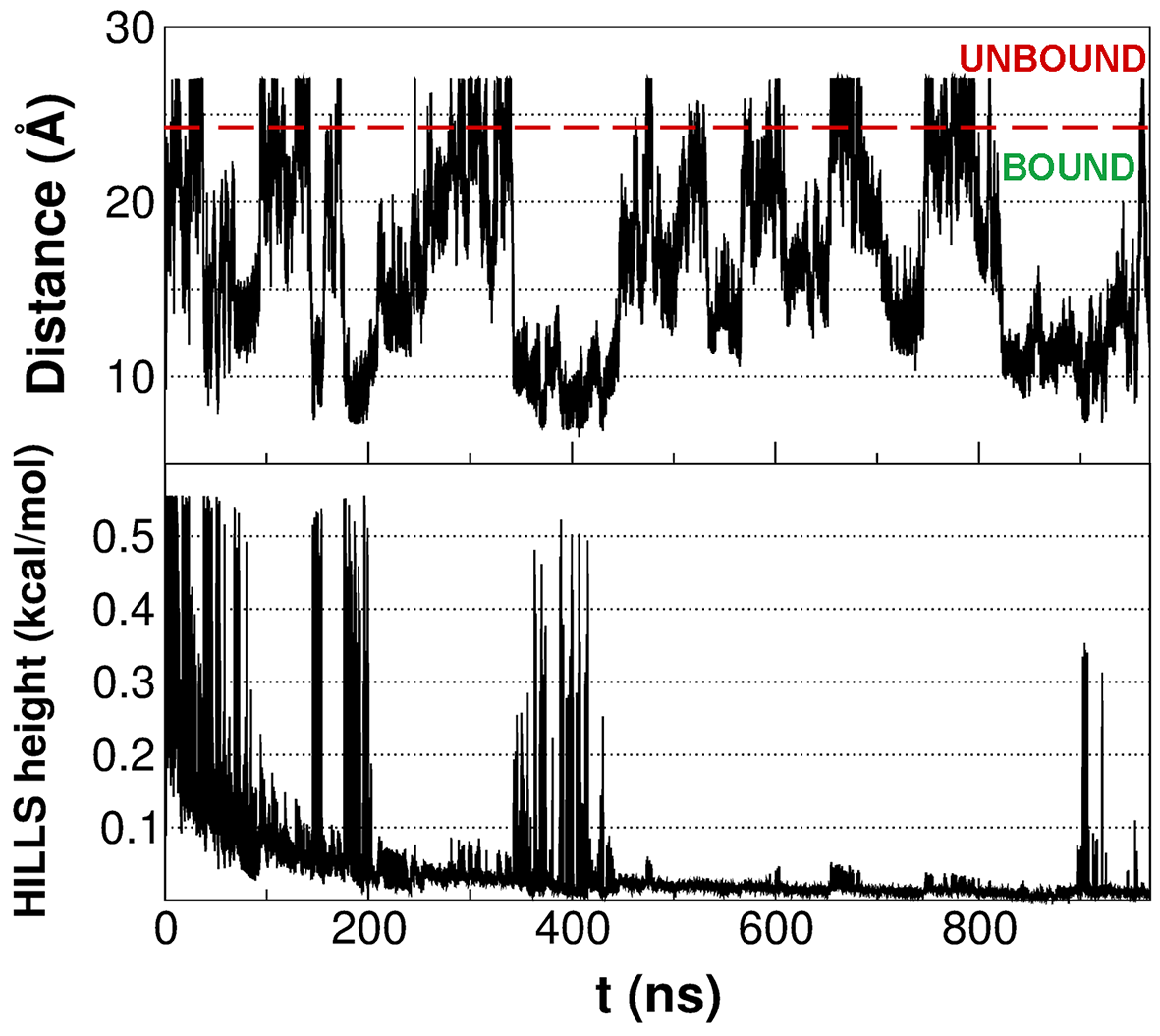
**

**Supplementary Figure S6.** (*Upper*) Plot of the distance CV along the metadynamics simulation on the **1**/[d(TGGGGT)]_4_ system. (*Lower*) Plot of the Gaussian height added to the system along the metadynamics simulation. The plot shows several recrossing events between bound and unbound states. These events lead to a quantitatively well-characterized FES with an accurate estimate of the absolute DNA-ligand binding free energy (see Supplementary Figure S7).

**
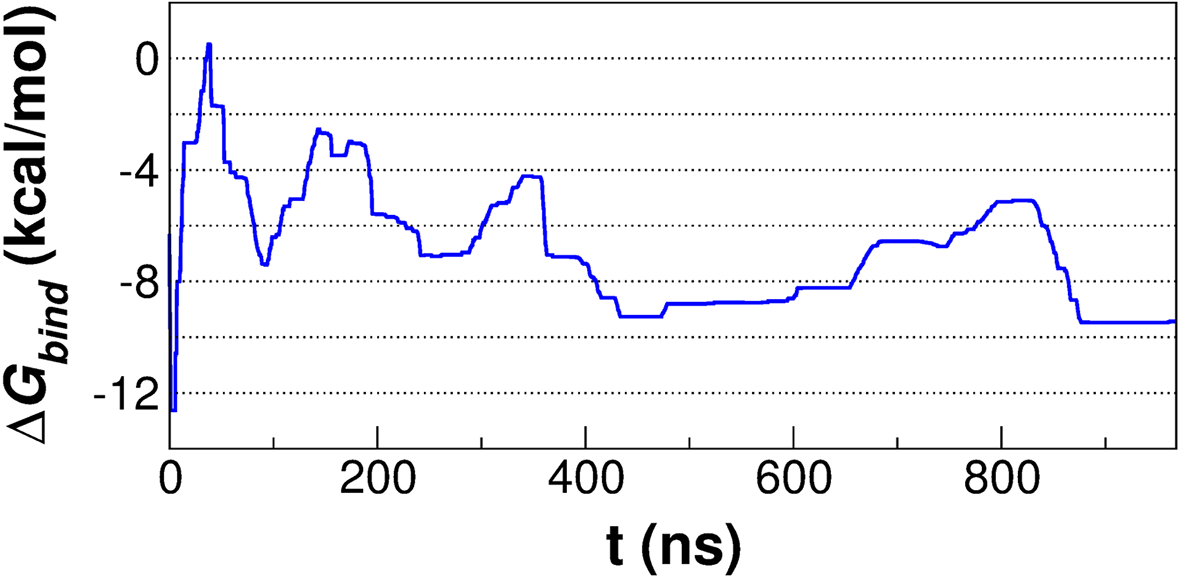
**

**Supplementary Figure S7.** Convergence of the absolute **1**/[d(TGGGGT)]_4_ binding free energy. The ΔG*_bind_* is calculated every 100 ps along the metadynamics simulation to assess the convergence. The bound state is defined at 7.0 ≤ *d* ≤ 14.0 Å, and the unbound one at 24.0 ≤ *d* ≤ 26.0 Å. The estimate of ΔG*_bind_* is −9.4 ± 1.4 kcal/mol. The uncertainty is calculated as the standard deviation from the asymptotic value of the absolute DNA-ligand binding free energy obtained from the last 450 ns of simulation.

**SUPPLEMENTARY TABLES**

Supplementary Table S1

| **List of atoms used to define the distance (*d*) and torsion (*φ*) CVs used in the metadynamics simulations. Distance CV is defined as the distance between the center of mass of groups of atoms.** | | | |
| --- | --- | --- | --- |
| **CV type** | **Atom** | | **DNA (D), ligand (L)** |
| *d* | CMR* | | L |
| *d* | Middle K^+^ ion | | D |
| *φ* | 5’ K^+^ ion | | D |
| *φ* | 3’ K^+^ ion | | D |
| *φ* | C6 of the benzo[*d*]thiazol ring | | L |
| *φ* | C7 of the 2*H*-chromen-2-one ring | | L |
|  | | | |
| **List of atoms used to define the projection on axis (POA) and distance from axis (DFA) CVs.** | | | |
| **CV type** | | **Atom** | **DNA (D), ligand (L)** |
| POA, DFA | | 5’ K^+^ ion | D |
| POA, DFA | | 3’ K^+^ ion | D |
| POA, DFA | | CMR* | L |
|  | |  |  |
| **List of atoms used to define the torsion CV (*ψ*) accounting for the orientation of the (N-(2-hydroxyethyl)piperazinyl)methyl tail of 1 relative to the G-quadruplex axis.** | | | |
| **CV type** | | **Atom** | **DNA (D), ligand (L)** |
| *ψ* | | 5’ K^+^ ion | D |
| *ψ* | | 3’ K^+^ ion | D |
| *ψ* | | C1 of the 2-hydroxyethyl group | L |
| *ψ* | | C of the methylene bridge | L |
| *CMR is the center of the mass of the ligand 3-(benzo[*d*]thiazol-2-yl)-2*H*-chrom-en-2-one ring | | | |

**SUPPLEMENTARY MOVIE S1**

Binding mechanism of compound **1** to DNA G-quadruplex under the action of metadynamics. The ligand explores all the potential binding sites on DNA with several recrossing events between the bound and unbound states. Such sampling leads to a well-converged binding free energy surface and an accurate estimate of the absolute DNA-ligand binding free energy.
